# Seasonality of respiratory syncytial virus and its association with meteorological factors in 13 European countries, week 40 2010 to week 39 2019

**DOI:** 10.2807/1560-7917.ES.2022.27.16.2100619

**Published:** 2022-04-21

**Authors:** You Li, Xin Wang, Eeva K Broberg, Harry Campbell, Harish Nair, European RSV Surveillance Network, European RSV Surveillance Network, Monika Redlberger-Fritz, Hanne-Dorthe Emborg, Ramona Trebbien, Joanna Ellis, Jamie Lopez Bernal, Olga Sadikova, Liidia Dotsenko, Kostas Danis, Sophie Vaux, Silke Buda, Janine Reiche, Anna Papa, Lisa Domegan, Linda Dunford, Adam Meijer, Anne C Teirlinck, Karol Szymański, Lidia B Brydak, Ana Paula Rodrigues, Raquel Guiomar, Jim McMenamin, Louise Shaw Primrose, Maja Sočan, Katarina Prosenc, Jesús Oliva, Inmaculada Casas, Simon Cottrell, Catherine Moore

**Affiliations:** 1 School of Public Health, Nanjing Medical University, Nanjing, China; 2 Centre for Global Health, Usher Institute, University of Edinburgh, Edinburgh, United Kingdom; 3 European Centre for Disease Prevention and Control, Stockholm, Sweden; 4 Members of the network have been listed under Collaborators and at the end of this article.

**Keywords:** Respiratory syncytial virus, Europe, seasonality, temperature, humidity

## Abstract

**Background:**

Respiratory syncytial virus (RSV) is the predominant cause of clinical pneumonia among infants and young children, often peaking during the winter months in temperate regions.

**Aim:**

To describe RSV seasonality in 13 European countries and examine its association with meteorological factors.

**Methods:**

We included weekly RSV seasonality data from 13 European countries between week 40 2010 and week 39 2019. Using local weighted regression method, we modelled weekly RSV activity with meteorological factors using data from the 2010/11 to the 2017/18 season. We predicted the weekly RSV activity of the 2018/19 season across 41 European countries and validated our prediction using empirical data.

**Results:**

All countries had annual wintertime RSV seasons with a longitudinal gradient in RSV onset (Pearson’s correlation coefficient, r = 0.71, 95% CI: 0.60 to 0.80). The RSV season started 3.8 weeks later (95% CI: −0.5 to 8.0) in countries in the eastern vs western parts of Europe, and the duration ranged from 8–18 weeks across seasons and countries. Lower temperature and higher relative humidity were associated with higher RSV activity, with a 14-day lag time. Through external validation, the prediction error in RSV season onset was −2.4 ± 3.2 weeks. Similar longitudinal gradients in RSV onset were predicted by our model for the 2018/19 season (r = 0.45, 95% CI: 0.16 to 0.66).

**Conclusion:**

Meteorological factors, such as temperature and relative humidity, could be used for early warning of RSV season onset. Our findings may inform healthcare services planning and optimisation of RSV immunisation strategies in Europe.

## Introduction

Respiratory syncytial virus (RSV) represents a substantial burden of disease globally among young children [1] and older adults [2]. A recent study among seven European countries suggests that 57–72% of respiratory tract infection-associated hospitalisations in children under 5 years of age were due to RSV and the proportion was even higher (62–87%) in children under 1 year [3]. Despite the substantial burden of RSV in children, there is no licensed vaccine for RSV. Currently, the only licensed RSV prevention product is palivizumab, a short-acting monoclonal antibody (mAb), which needs to be administered to infants every month for a total of 5 months from the onset of the RSV season. Palivizumab is very expensive (EUR 3,400–5,600 per child [4]) and hence is administered mainly to high-risk infants and mostly in high-income countries. Nonetheless, there are over 40 candidate RSV prophylactic products at different phases of clinical trials up to March 2020 [5]. Recent results from clinical trials of a long-acting mAb, nirsevimab [6], and a maternal vaccine, ResVax (Novavax) [7], supported these passive immunisation strategies for preventing RSV disease in young infants. However, the duration of protection conferred by these prophylactics is limited (up to 5 months) and thus the seasonality of RSV needs to be accounted for when planning RSV immunisation strategies.

Globally, RSV causes annual seasonal epidemics in most areas, including both temperate and tropical regions [8,9] and meteorological factors including temperature and humidity have been reported to be associated with RSV activity [8]. A study of 15 European countries showed that RSV season started at around week 49 (early December) and lasted 8 to 24 weeks; RSV detections peaked later (r = 0.56; p = 0.04) and seasons lasted longer with increasing latitude (r = 0.57; p = 0.03) [10]. However, there is a lack of studies that report on the association between RSV seasonality and meteorological factors in Europe. In the present study, we aimed to model the RSV seasonality in 13 European countries with various meteorological factors using multi-season surveillance data.

## Methods

### Data sources

We defined the start of a season as the week 40 of a given year and the end of that season as the week 39 of the next year. We included country-specific data on laboratory-confirmed RSV cases between the 2010/11 season and the 2018/19 season from the RSV surveillance dataset by the European Centre for Disease Prevention and Control (ECDC). These data were reported weekly to ECDC through the European Influenza Surveillance Network (EISN), as detailed elsewhere [10,11]. There was no specific testing eligibility for RSV and the criteria for RSV testing could vary substantially by country and by type of RSV surveillance [10,11]. Clinicians either tested patients with influenza-like illness or acute respiratory infection, or ordered testing based on their own clinical judgement. 

Sentinel surveillance used random or systematic sampling approaches whereas non-sentinel surveillance was mostly based on clinician’s judgement. Data from different surveillance systems, i.e. sentinel surveillance and non-sentinel surveillance, of the same country were regarded as separate data sources because of the heterogeneity in the population and testing criteria. For the present study, we considered eligibility for inclusion of RSV data on a surveillance season basis. For each season per surveillance system data source, we included RSV data if more than 25 RSV-positive samples were reported and the number of weeks with missing data was no more than five, i.e. ≤ 10% of the season.

Country-specific meteorological data from the same study period were extracted from the Global Surface Summary of the Day dataset provided by the US National Centers for Environmental Information (NCEI), via the R package ‘GSODR: Global Surface Summary of the Day Weather Data Client’ [12]. For each country, all weather stations available nationwide from the study period were included. The variables extracted included temperature, relative humidity, wind speed, precipitation and dew point, available as daily average values.

### Description of respiratory syncytial virus seasonality

In order to account for delay in reporting, e.g. during national holidays, we calculated 3-week moving average of RSV-positive samples per week. Spline interpolation was used to impute missing numbers of RSV cases, where applicable, before applying the 3-week moving average. We then calculated annual percentages per week for each season and surveillance system data source, separately. The reported annual percentage represents the strength of RSV activity and annual percentages together added up to 100% per season and surveillance system data source.

We used the same approach as previously reported to define RSV ‘epidemic weeks’ [8]. This was done by identifying the minimum number of weeks with highest RSV activity required to jointly account for at least 75% of the annual RSV cases, with each of these weeks defined as an epidemic week; the epidemic weeks might be non-consecutive to allow for the occasional off-season small uptick in the RSV activity. The onset of an RSV season was defined as the first of the two consecutive epidemic weeks in each season. The offset of an RSV season was defined as the first epidemic week that was followed by three consecutive non-epidemic weeks in each season. The duration of an RSV season was defined as the interval between the onset and the offset of that RSV season. The coordinates, i.e. longitude and latitude, of country’s centroid were used for assessing the correlation between longitude/latitude and RSV seasonality. A country is defined as being in the eastern part of Europe if the longitude of the country’s centroid is > 20° and in the western part if the centroid longitude is ≤ 20°.

### Seasonality model

We developed a local weighted regression model with data-driven selection of model predictors, i.e. meteorological factors, and model parameters (for degree of smoothness). Details of the seasonality model are in Supplement S1. Briefly, we considered different combinations of meteorological parameters and different time lags (0, 7, 14, 21, 35 and 42 days). Through a modelling selection process, we selected the model of mean-centred temperature and relative humidity with a time lag of 14 days as the main model (detailed model selection results are in Supplementary Tables S1–S3). The RSV seasonality data from the season 2010/11 to the season 2017/18 were used as the training and internal validation dataset for building the model. Considering the heterogeneity of criteria of RSV testing in different surveillance systems, we repeated the same modelling exercise excluding those surveillances that relied on clinicians’ judgement for RSV testing as sensitivity analysis.

Using our main model, we predicted the weekly activity of RSV in 41 European countries for the season 2018/19 with country-specific meteorological data as the input and we determined the critical values of temperature and relative humidity that indicated RSV onset for each country. We further validated our prediction using the observed data from the 2018/19 season (which were not used for modelling) by comparing the predicted RSV onset season with the observed RSV onset season.

All data analyses and visualisation were conducted using the R software (version 3.6.1) [13].

## Results

### Respiratory syncytial virus seasonality

**Figure 1:**
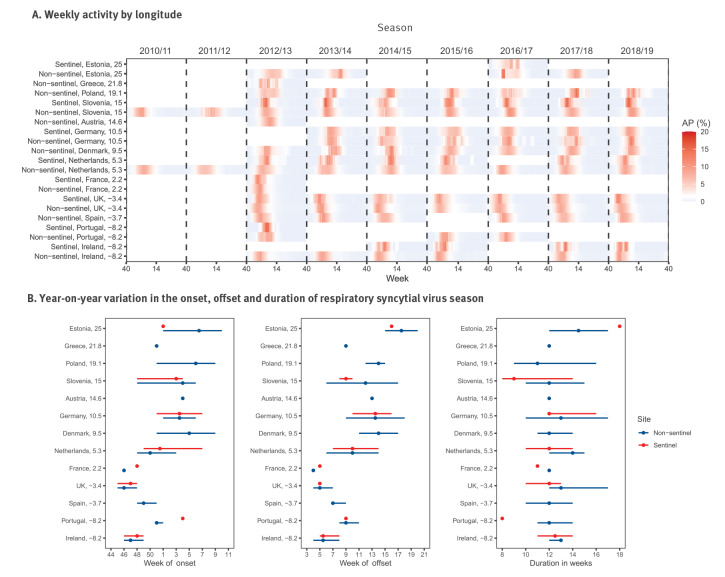
Respiratory syncytial virus seasonality in 13 European countries, week 40 2010–week 39 2019

AP: annual percentage; RSV: respiratory syncytial virus; UK: United Kingdom.

We included 100 seasons from 21 surveillance systems of 13 countries. The number of RSV-positive samples per season and surveillance system ranged between 27 and 15,315 (Table). All the countries included had annual RSV seasons with a longitudinal gradient observed in RSV onset (Pearson’s correlation coefficient, r = 0.71, 95% confidence interval (CI): 0.60 to 0.80; Figure 1); the average RSV onset of countries in the east of Europe (defined by longitude > 20° of country’s centroid) was 3.8 weeks later (95% CI: −0.5 to 8.0) than those in the western part of Europe. No correlation was observed between RSV onset and latitude (r = −0.06, 95% CI: –0.25 to 0.14; Supplementary Figure S1). A year-on-year variation of ± 4 weeks in the RSV onset was observed in most countries, with the exception of Slovenia, Estonia and Denmark that showed higher variations; similar variation (± 4 weeks) was observed in the RSV offset (Figure 1). Duration of RSV season ranged between 8 and 18 weeks across seasons and countries.

**Table:** Case definitions, number of RSV seasons and RSV-positive samples by country and surveillance system in 13 European countries, week 40 2010–week 39 2019

Country	Surveillance system^a^	Case definition for sampling	Number of seasons	RSV-positive samples per season	
				Median	Range
Austria	Non-sentinel	ILI	1	861	NA
Denmark	Non-sentinel	2012–15: ARI/ILI^b^ 2015–19: clinical judgement^c^	7	2,619	45–4,507
Estonia	Non-sentinel	ARI/ILI	4	484	177–644
Estonia	Sentinel	ILI	1	52	NA
France	Non-sentinel	Clinical judgement	1	9,074	NA
France	Sentinel	ILI	1	382	NA
Germany	Non-sentinel	ARI	6	132	56–285
Germany	Sentinel	ARI	6	295	220–430
Greece	Non-sentinel	ILI	1	36	NA
Ireland	Non-sentinel	Clinical judgement	6	965	684–1,572
Ireland	Sentinel	ILI	4	30	27–32
Netherlands	Non-sentinel	Clinical judgement	9	1,889	1,390–2,729
Netherlands	Sentinel	ARI/ILI	6	47	32–104
Poland	Non-sentinel	ILI	7	135	35–464
Portugal	Non-sentinel	ARI/ILI/clinical judgement	3	630	79–665
Portugal	Sentinel	Influenza-negative cases	1	38	NA
Slovenia	Non-sentinel	Clinical judgement	9	1,379	666–1,538
Slovenia	Sentinel	ILI	7	49	31–60
Spain	Non-sentinel	Clinical judgement	6	3,262	1,747–4,350
United Kingdom	Non-sentinel	ARI/ILI	7	11,014	4,744–15,315
United Kingdom	Sentinel	ILI	7	211	162–327

ARI: acute respiratory infection; ILI: influenza-like illness; NA: not applicable; RSV: respiratory syncytial virus.

^a^ Sentinel surveillance is defined by a system that is set up for surveillance as a primary goal; non-sentinel surveillance is defined by a system that is not set up for surveillance.

^b^ Data from the national laboratory at Statens Serum Institut, Denmark.

^c^ Data from all clinical microbiological laboratories in Denmark.

Sentinel surveillance is defined by a system that is set up for surveillance as a primary goal; non-sentinel surveillance is defined by a system that is not set up for surveillance. Annual percentage was calculated to represent strength of RSV activity; annual percentages together added up to 100% for each season. The value next to each country denotes the longitude of each country’s centroid.

### Association between respiratory syncytial virus activity and meteorological factors

**Figure 2:**
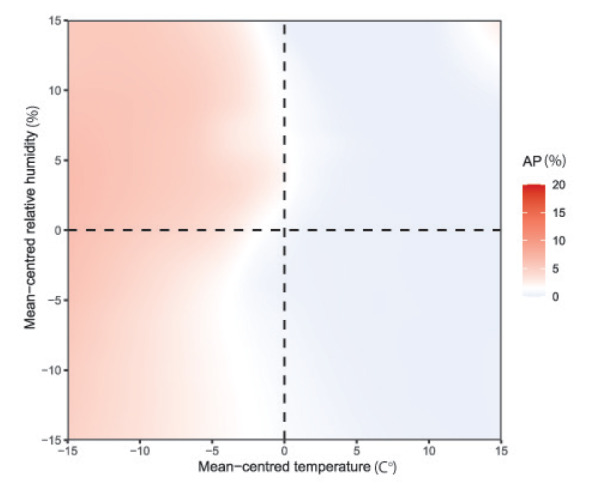
Association between meteorological factors and respiratory syncytial virus activity from 13 European countries, week 40 2010–week 39 2019

**Figure 3:**
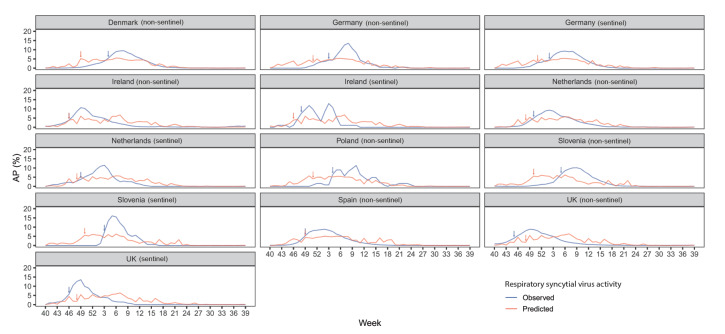
Comparison of respiratory syncytial virus activity between model prediction and observation in 8 European countries, 2018/19 season

AP: annual percentage; UK: United Kingdom.

In the main model with a 14-day lag time, we found that lower temperature and higher relative humidity (vs the corresponding annual average value) were associated with higher RSV activity (Figure 2). A sensitivity analysis that excluded those surveillance systems that relied on clinicians’ judgement for RSV testing had consistent findings (Supplementary Figure S2: Association between meteorological factors and respiratory syncytial virus activity from sensitivity analysis). The Pearson’s correlation coefficient was –0.61 (95% CI: –0.63 to –0.59) between temperature and RSV activity, and was 0.40 (95% CI: 0.37 to 0.42) between relative humidity and RSV activity. The critical values of temperature and relative humidity that indicated RSV onset differed by country, ranging from –19°C to 14°C and from 67% to 96%, respectively. Supplementary Table S4 shows the critical values of temperature and relative humidity. When comparing the externally predicted activity of RSV with the observed activity across eight countries that had available data in the 2018/19 season, we found that the prediction error in the RSV season onset was –2.4 ± 3.2 weeks and that the model prediction was more conservative than the observed data regarding the amplitude of peak RSV activity (Figure 3).

AP: annual percentage.

The colour scale refers to the predicted weekly percentage of respiratory syncytial virus cases in annual cases.

Sentinel surveillance is defined by a system that is set up for surveillance as a primary goal; non-sentinel surveillance is defined by system that is not set up for surveillance. The observed respiratory syncytial virus activity is presented in blue and the predicted respiratory syncytial virus activity is presented in red. Arrows denote the respiratory syncytial virus season onset.

**Figure 4:**
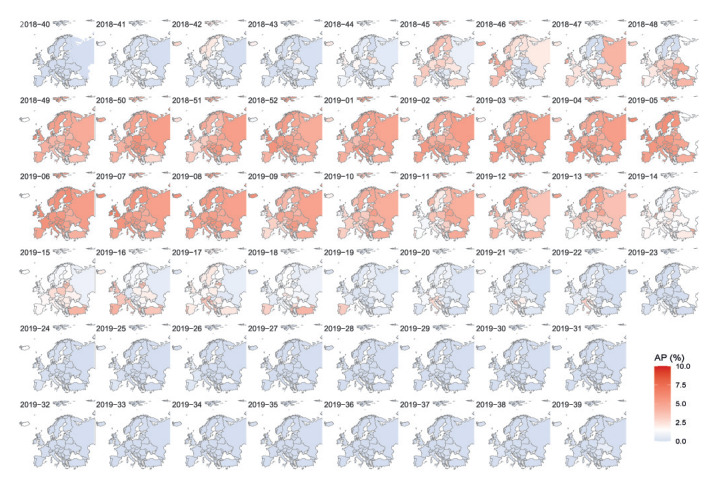
Model-predicted weekly percentage of respiratory syncytial virus cases of all annual cases in 41 European countries, 2018/19 season

In the model-predicted weekly RSV activity for the 2018/19 season across 41 European countries, available in Figure 4, a similar longitudinal gradient was observed in the RSV onset (r = 0.45, 95% CI: 0.16 to 0.66).

AP: annual percentage.

The colour scale refers to the predicted weekly percentage of respiratory syncytial virus cases of all annual cases. The number in each map panel denotes the year and week number.

## Discussion

To the best of our knowledge, this is the first study that has modelled RSV seasonality with meteorological factors using multi-season data across Europe. All 13 European countries in our study showed clear annual wintertime RSV seasons with a west-to-east gradient observed in the RSV onset; countries in the eastern part of Europe had a later RSV season (ca 4 weeks) than those in the western part of Europe. We found that lower temperature and higher relative humidity were associated with higher RSV activity, with a time lag of 14 days. Through external validation, the prediction error in the RSV season onset of our model was –2.4 ± 3.2 weeks. A delayed RSV onset in the east of Europe was also seen in our model predictions for RSV seasonality in 41 European countries for the 2018/19 season.

Our findings on the RSV seasonality in European countries were broadly similar to those reported earlier by Broberg et al. [10] that included RSV data from 2010–16 with less strict criteria and different definitions for RSV season, although they report different findings in the latitudinal/longitudinal patterns of the RSV onset. In their study [10], both latitudinal and longitudinal gradients in the RSV onset were identified; in contrast, our study identified a longitudinal but not a latitudinal gradient in the RSV onset. The absence of a latitudinal gradient in Europe observed in our study was different to our previous findings from a global perspective; a consistently clear latitudinal gradient in the global RSV onset was reported in studies using different data sources [8,14,15]. Nonetheless, one of these global studies [8] found that within Europe, only a longitudinal gradient was observed in the RSV onset (r = 0.46) and the average difference in the onset between the western and eastern parts of Europe was 0.8 months, which was very similar to our findings (r = 0.71 and the difference in the onset = 3.8 weeks). The longitudinal and non-latitudinal gradient in the RSV onset observed in our study highlights the unique pattern of RSV timing in Europe (vs globally). Similarly, only a significant longitudinal gradient was observed for influenza [16]. One possible explanation for the different longitudinal/latitudinal gradients observed in Europe from that observed globally is that, within Europe, the climate – as measured by meteorological metrics, e.g. temperature and humidity – varied more by longitude than latitude. This explanation was supported by our model predictions among 41 European countries, which replicated the longitudinal gradients using temperature and humidity information. The observed longitudinal gradient in RSV onset could be relevant to countries with a large longitudinal span such as Russia, since a single immunisation programme for the whole country might not be optimal.

Based on the data-driven model selection process, we found that the model of temperature and relative humidity with a lag of 14 days (approximately two times the serial interval of RSV [17]) had the best external predictability. This potentially has important implications for early warning of RSV seasons for countries in Europe. For example, upon observations that temperature and/or relative humidity are reaching the critical values (in Supplementary Table S4: Critical values of temperature and relative humidity), countries could prepare for a possible surge in the hospital beds needs – especially for paediatric wards – in the coming 2 weeks. RSV transmission in the community is likely to happen earlier than that after accounting for the lags in attainment of healthcare and subsequent confirmation of infection. Our model results are also relevant to the RSV immunisation strategies, including both maternal vaccines and passive monoclonal antibody immunisation for infants. A recent modelling study across 52 low- and middle-income countries reported that a seasonal schedule for passive RSV immunisations could be substantially more efficient than a year-round approach without losing much effectiveness [18]. However, our model prediction was overall more conservative with milder peak RSV activity being predicted than the observed RSV peak; one of the possible reasons for this was increased indoor crowding during the peak season, which was not accounted for by the model due to the scarcity of relevant data.

Our study is not without limitations. Firstly, there was high heterogeneity among different surveillance systems of the included countries in terms of population (age), setting (primary care vs secondary care), sampling strategy, eligibility for testing and testing method. Secondly, all of the 13 included countries were European Union/European Economic Area members and might not fully represent all countries in Europe. Only 1 year of data was available for four included countries and thus we were not able to describe the year-on-year variation in RSV season for these countries. Thirdly, we lacked the data granularity to explore RSV seasonality on a sub-country level or by age group, e.g. paediatric vs elderly populations. Fourthly, our model only accounted for meteorological factors; other factors including human behaviours could also drive viral transmission although some of these were associated with meteorological factors, e.g. more indoor crowding in cold weather. Since the beginning of the COVID-19 pandemic, a near cessation of RSV transmission during periods of movement restrictions and physical distancing has been observed. However, there have been reports on out-of-season RSV activity in several countries, possibly driven by the relaxation of non-pharmaceutical interventions as well as the growing population susceptibility [19,20]. Our current modelling framework was not designed to account for these factors as our data were all collected before the COVID-19 pandemic. Finally, we lacked the data to be able to explore the role of virus-virus interaction in shaping RSV seasonality; a recent modelling study in Scotland supported positive interactions between RSV and human metapneumovirus [21].

## Conclusions

Results from our study provide a baseline picture of RSV seasonality and its association with meteorological factors in the pre-RSV-vaccination and pre-COVID-19 era in Europe. With a few RSV prophylactic vaccine candidates on the horizon, our findings on RSV seasonality in Europe along with the prediction model may inform the timing of RSV immunisations as well as seasonal healthcare services planning.

## European RSV Surveillance Network

Monika Redlberger-Fritz (Center for Virology, Medical University Vienna, Austria), Hanne-Dorthe Emborg and Ramona Trebbien (Statens Serum Institut, Denmark), Joanna Ellis and Jamie Lopez Bernal (Public Health England, England, UK), Olga Sadikova and Liidia Dotsenko (Estonian Health Board, Estonia), Kostas Danis and Sophie Vaux (Santé publique France, National public health institute, France), Silke Buda and Janine Reiche (Robert Koch Institute, Germany), Anna Papa (University of Thessaloniki, Greece), Lisa Domegan (Health Protection Surveillance Centre, Ireland), Linda Dunford (University College Dublin, Ireland), Adam Meijer and Anne C Teirlinck (National Institute for Public Health and the Environment [RIVM], Netherlands), Karol Szymański (National Institute of Public Health - National Institute of Hygiene, Poland), Lidia B Brydak (National Influenza Centre, Poland), Ana Paula Rodrigues and Raquel Guiomar (National Institute of Health Doutor Ricardo Jorge, Portugal), Jim McMenamin and Louise Shaw Primrose (National Health Service National Services Scotland, Scotland, UK), Maja Sočan (National Institute of Public Health, Slovenia), Katarina Prosenc (National Institute for Health, Environment and Food, Slovenia), Jesús Oliva and Inmaculada Casas (Instituto de Salud Carlos III, Spain), and Simon Cottrell and Catherine Moore (Public Health Wales, Wales, UK).

## Supplementary Data

845000 Supplement
