# Supplementary material for: Seasonality of respiratory syncytial virus and its association with meteorological factors in 13 European countries, week 40 2010 to week 39 2019
Source: Euro Surveill. 2022 Apr 21;27(16):2100619. doi: 10.2807/1560-7917.ES.2022.27.16.2100619 (PMC9027150; doi:10.2807/1560-7917.ES.2022.27.16.2100619)
Supplement: Supplement [file 21-00619_LI_Supplement.pdf]

This supplementary material is hosted by *Eurosurveillance* as supporting information alongside the article “**Seasonality of respiratory syncytial virus and its association with meteorological factors in 13 European countries, week 40 2010 to week 39 2019**”, on behalf of the authors, who remain responsible for the accuracy and appropriateness of the content. The same standards for ethics, copyright, attributions and permissions as for the article apply. Supplements are not edited by *Eurosurveillance* and the journal is not responsible for the maintenance of any links or email addresses provided therein.

Contents

Supplement S1: Details on the prediction model 3
Data 3
Model selection 3
Step 1. One-predictor model 3
Table S1. Average square error by leave-one-country-season-out cross-validation among one-predictor candidate models 3
Step 2. Two-predictor model 4
Table S2. Average square error by leave-one-country-season-out cross-validation among two-predictor candidate models 4
Step 3. Three-predictor model 4
Table S3. Average square error by leave-one-country-season-out cross-validation among three-predictor candidate models 4
Table S4. Critical values of temperature and relative humidity indicating the onset of respiratory syncytial virus season (in the next 14 days) for 41 European countries, based on the prediction for the season 2018/19 5
Figure S1. Respiratory syncytial virus seasonality in 2010–2019 in 13 European countries by latitude 7
Figure S2. Association between meteorological factors and respiratory syncytial virus activity from sensitivity analysis that excluded those surveillances that relied on clinicians' judgement for testing 8
Reference 9

Supplement S1: Details on the prediction model

Data

Daily meteorological data of each country were extracted from the US National Oceanic and Atmospheric Administration (NOAA) via the R package GSODR [1]. For each country, all weather stations available nationwide from 2010 to 2019 were included. The variables that were directly extracted included daily average temperature, relative humidity, wind speed, dew point and precipitation. In addition, we calculated the daily absolute humidity from temperature and relative humidity using the following formula:

$$AH = \frac{6.112 \times e^{\frac{17.67 \times T}{243.5+T}} \times RH \times 2.1674}{T + 273.15}$$

where AH is absolute humidity as grams/m^3; RH is relative humidity as %; and T is temperature in Celsius [2]. For each variable above, we calculated the mean-centred values as the difference between the daily value and the annual average value, which reflects the relative change of each meteorological measure. We considered all the variables and their mean-centred measures as candidate predictors.

In order to match with the weekly aggregated RSV counts data, we aggregated the daily measures of all candidate predictors by week. We also shifted these measures by 0, 7, 14, 21, 28, 35 and 42 days, defined a priori, to allow for a time lag between these predictors and RSV activity.

Model selection

Only data in 2010–2018 were used for the model as the training and validation dataset (through leave-one-out cross-validation); data from the season 2018/2019 were used as the testing dataset. We used LOESS model with a data-driven specification of its parameter "span" (range: 0–1), which defined the smoothness of the fitted curve [3]. Model selection was based on assessing the average square error per "surveillance-season" by leave-one-"country-season"-out cross-validation. Note that one country might have two surveillance systems (i.e. sentinel surveillance and non-sentinel surveillance). For each candidate model, the parameter span was selected that minimised the average square error. The detailed steps of the model selection are described below:

Step 1. One-predictor model

We first assessed the performance of one-predictor model with two specific objectives: first, we needed to decide whether to use identity measure or mean-centred measure for the predictors; and secondly, we needed to select a subset of predictors for the subsequent multiple-predictor model, which is more computer-intensive. The model selection results are in **Table S1**. The mean-centred measure was found to be predominantly favourable than the identity measure across different predictors. Temperature, relative humidity, dew point and absolute humidity had smaller prediction errors and thus were selected for the two-predictor model selection.

Table S1. Average square error by leave-one-country-season-out cross-validation among one-predictor candidate models

Candidate	Lag=0d	Lag=7d	Lag=14d	Lag=21d	Lag=28d	Lag=35d	Lag=42d
**TEMPc***	245.99	238.50	**237.71**	243.81	255.64	271.42	288.48
**RHc**	376.12	362.51	347.35	333.60	322.77	315.56	**311.00**
**WDSPc**	**405.32**	406.20	407.89	410.56	413.28	416.04	419.33
**DEWPc**	256.39	**255.83**	261.61	272.62	286.60	302.75	319.09
**PRCPc**	434.19	434.72	434.90	**432.52**	433.13	433.49	433.22
**AHc**	**265.44**	266.01	273.12	284.62	298.88	315.93	333.52
**TEMP**	258.75	251.27	**251.03**	255.80	266.57	282.20	300.85
**RH**	395.62	382.35	367.40	353.04	341.80	332.08	**325.25**
**WDSP**	**418.95**	419.66	420.52	421.68	422.50	423.42	424.42
**DEWP**	268.25	**267.14**	271.57	280.25	292.40	307.65	323.96
**PRCP**	433.10	433.91	434.17	**432.17**	433.14	433.88	434.12
**AH**	276.07	**275.46**	280.33	289.18	301.07	316.26	332.79

* The letter "c" denotes "mean-centred".
For each candidate model, value in bold and underline denotes minimum prediction error.

TEMP = temperature; RH = relative humidity; WDSP = wind speed; DEWP = dew point; PRCP = precipitation; AH = absolute humidity

Step 2. Two-predictor model

All combinations of the selected predictors were considered for the two-predictor model. The model selection results are in **Table S2**. A further reduction in the prediction error was observed in two-predictor models than one-predictor models. The model with mean-centred temperature and relative humidity had the best performance.

Table S2. Average square error by leave-one-country-season-out cross-validation among two-predictor candidate models

Candidate	Lag=0d	Lag=7d	Lag=14d	Lag=21d	Lag=28d	Lag=35d	Lag=42d
**TEMPc+RHc***	234.96	224.16	**<u>218.26</u>**	218.61	225.09	235.80	245.92
**TEMPc+DEWPc**	248.88	239.19	233.02	**<u>232.16</u>**	235.47	242.78	252.01
**TEMPc+AHc**	246.06	235.15	228.84	**<u>228.35</u>**	233.91	241.65	249.53
**RHc+DEWPc**	235.03	224.20	**<u>218.28</u>**	219.22	224.87	234.74	245.14
**RHc+AHc**	242.61	231.05	**<u>225.75</u>**	226.64	233.66	244.05	255.55
**DEWPc+AHc**	**<u>260.12</u>**	261.46	263.02	272.53	283.95	299.09	317.01

* The letter "c" denotes "mean-centred".
For each candidate model, value in bold and underline denotes minimum prediction error.
TEMP = temperature; RH = relative humidity; DEWP = dew point; AH = absolute humidity

Step 3. Three-predictor model

Based on the results from Step 2, we considered two candidate models for the three-predictor model comparison (**Table S3**). However, the prediction errors of the three-predictor models were found to be consistently higher than those of the two-predictor models, indicating potential over-fitting. Therefore, we decided to select the two-predictor model with mean-centred temperature and relative humidity as the final model and the model with mean-centred relative humidity and dew point as the secondary model for sensitivity analyses.

Table S3. Average square error by leave-one-country-season-out cross-validation among three-predictor candidate models

Candidate	Lag=0d	Lag=7d	Lag=14d	Lag=21d	Lag=28d	Lag=35d	Lag=42d
**TEMPc+RHc+DEWPc***	**<u>435.99</u>**	452.92	534.80	531.83	461.94	452.59	446.49
**TEMPc+RHc+AHc**	441.35	477.06	437.76	473.62	**<u>399.70</u>**	433.35	438.63

* The letter "c" denotes "mean-centred".
For each candidate model, value in bold and underline denotes minimum prediction error.
TEMP = temperature; RH = relative humidity; DEWP = dew point; AH = absolute humidity

Table S4. Critical values of temperature and relative humidity indicating the onset of respiratory syncytial virus season (in the next 14 days) for 41 European countries, based on the prediction for the season 2018/19

	Main analysis		Sensitivity analysis	
**Country**	**Temperature (°C)**	**Relative humidity (%)**	**Temperature (°C)**	**Relative humidity (%)**
**Albania**	6.35	68.75	6.35	68.75
**Armenia**	−3.12	76.09	-1.39	84.27
**Austria**	−1.89	82.42	-1.89	82.42
**Belarus**	0.05	96.13	0.05	96.13
**Belgium**	4.11	90.30	4.11	90.3
**Bosnia and Herzegovina**	3.52	81.53	0.23	85.51
**Bulgaria**	0.53	82.86	0.53	82.86
**Croatia**	7.13	77.33	7.13	77.33
**Cyprus**	12.17	69.05	12.46	75.7
**Czech Republic**	−2.06	86.22	-2.06	86.22
**Denmark**	3.35	87.73	3.35	87.73
**Estonia**	−3.95	78.03	-5.87	92.93
**Finland**	−7.36	90.63	-6.08	91.06
**France**	8.87	85.47	5.9	92.33
**Georgia**	3.41	80.62	3.53	85.53
**Germany**	0.95	83.03	0.95	83.03
**Greece**	10.74	71.93	10.74	71.93
**Hungary**	0.22	87.50	0.22	87.5
**Iceland**	1.75	82.25	1.75	82.25
**Ireland**	8.38	87.34	8.38	87.34
**Italy**	11.11	78.74	11.11	78.74
**Latvia**	1.33	88.41	1.33	88.41
**Liechtenstein**	0.10	69.04	5.33	77.98
**Lithuania**	−3.30	92.16	-3.3	92.16
**Moldova**	−2.99	87.87	0.68	90.16
**Montenegro**	6.94	67.27	6.94	67.27
**Netherlands**	3.84	90.50	3.15	81.96
**Norway**	0.03	84.41	1.18	79.65
**Poland**	−0.49	90.24	-0.49	90.24
**Portugal**	14.26	77.55	13.31	80.66
**Romania**	−0.18	85.68	-0.18	85.68
**Russia**	−18.55	76.71	-18.55	76.71
**Serbia**	2.49	83.17	2.49	83.17
**Slovakia**	0.38	86.95	-2.48	86.7
**Slovenia**	3.64	83.38	3.64	83.38
**Spain**	11.03	72.93	10.73	75.24
**Sweden**	−0.53	87.22	-3.02	88.07
**Switzerland**	2.15	82.90	2.15	82.9
**Turkey**	4.13	74.57	4.13	74.57
**UK**	5.54	91.75	5.54	91.75
**Ukraine**	−1.67	91.21	-1.67	91.21

In sensitivity analysis, surveillance sites where RSV testing was ordered at clinicians’ discretion were excluded.

Figure S1. Respiratory syncytial virus seasonality in 2010–2019 in 13 European countries by latitude

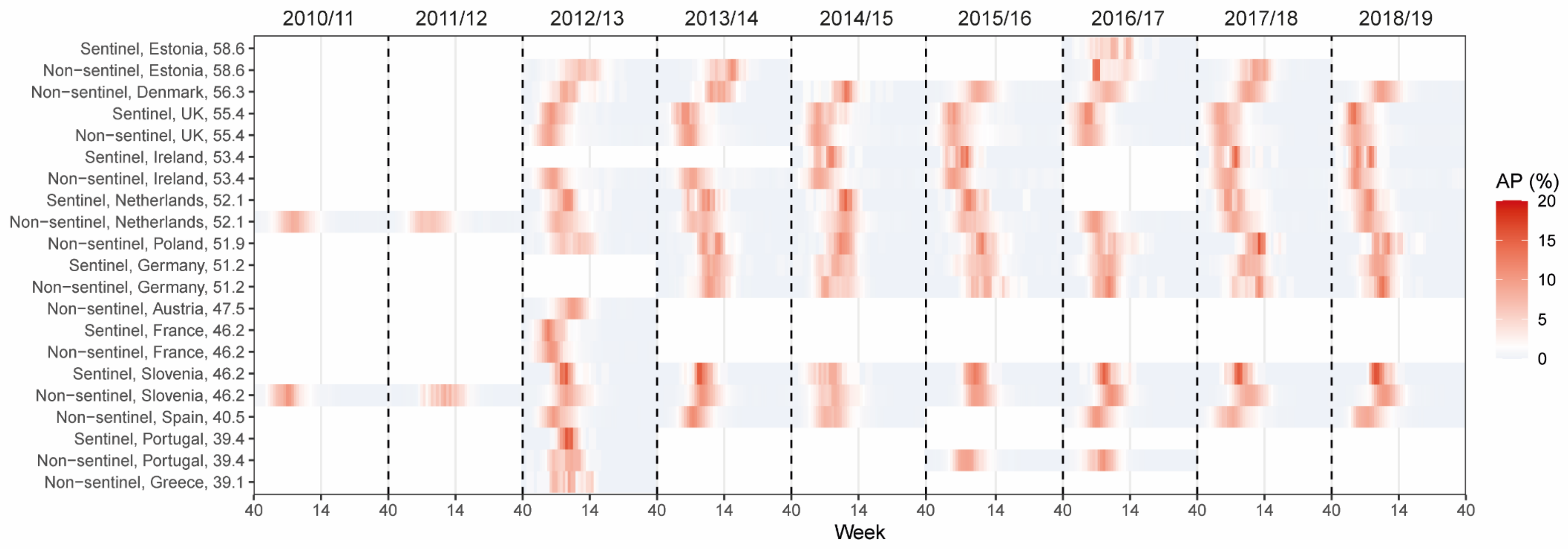

Annual percentage (AP) was calculated to represent strength of RSV activity and added up to 100% across weeks for each season. Sentinel surveillance is defined by a system that is set up for surveillance as primary goal. The value next to country denotes the latitude of country’s centroid.

Figure S2. Association between meteorological factors and respiratory syncytial virus activity from sensitivity analysis that excluded those surveillances that relied on clinicians' judgement for testing

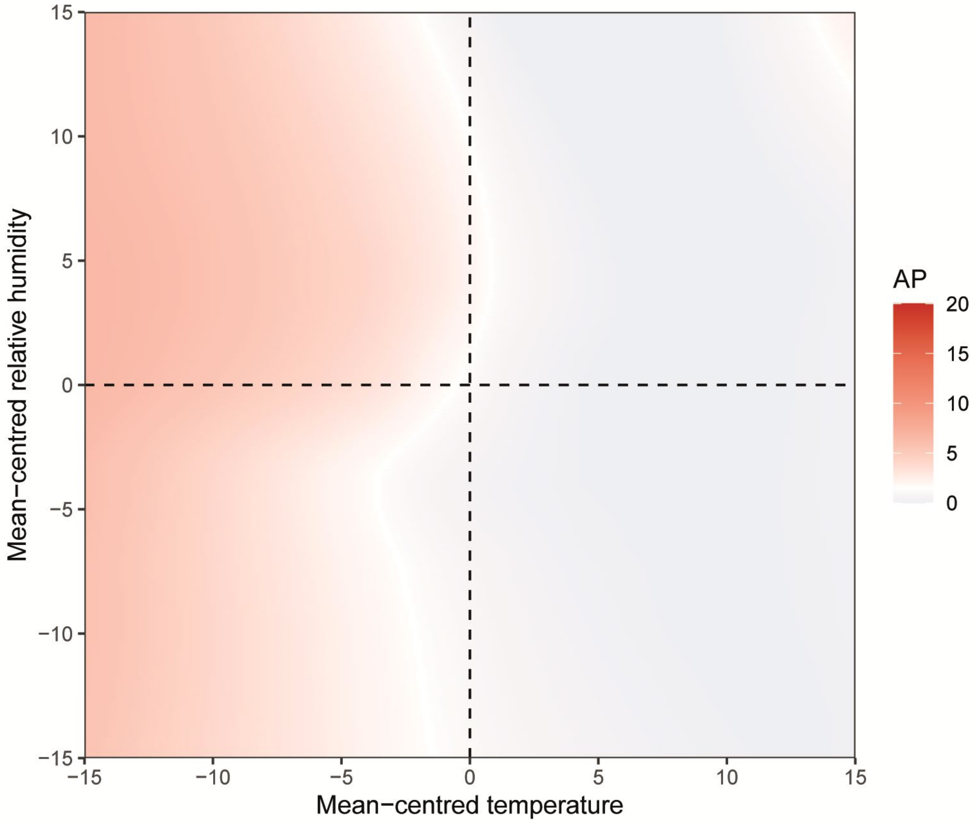

The colour scale refers to the predicted weekly percentage of respiratory syncytial virus cases in annual cases.

Reference

1. Sparks A, Hengl T, Nelson A. GSODR: Global Summary Daily Weather Data in R. Journal of open source software. 2017;2(10):177.
2. Bian Q. The nature of climate change-equivalent climate change model's application in decoding the root cause of global warming. Inter J Env Climate Change. 2019;9(12):801-22.
3. Li Y, Reeves RM, Wang X, Bassat Q, Brooks WA, Cohen C, et al. Global patterns in monthly activity of influenza virus, respiratory syncytial virus, parainfluenza virus, and metapneumovirus: a systematic analysis. The Lancet Global Health. 2019;7(8):e1031-e45.
